# Quantitative biodistribution of nanoparticles in plants with lanthanide complexes

**DOI:** 10.1038/s41598-023-47811-4

**Published:** 2023-12-05

**Authors:** H. Hou, Z. Xu, Y. S. Takeda, M. Powers, Y. Yang, K. Hershberger, Hailey Hanscom, S. Svenson, R. K. Simhadri, A. J. Vegas

**Affiliations:** 1 Division of Materials Science and Engineering, Boston University, Boston, MA USA; 2 Department of Chemistry, Boston University, Boston, MA USA; 3 Invaio Sciences, Cambridge, MA USA

**Keywords:** Biological techniques, Biological models, Nanobiotechnology, Nanoscale materials

## Abstract

The inefficient distribution of fertilizers, nutrients, and pesticides on crops is a major challenge in modern agriculture that leads to reduced productivity and environmental pollution. Nanoformulation of agrochemicals is an attractive approach to enable the selective delivery of agents into specific plant organs, their release in those tissues, and improve their efficiency. Already commercialized nanofertilizers utilize the physiochemical properties of metal nanoparticles such as size, charge, and the metal core to overcome biological barriers in plants to reach their target sites. Despite their wide application in human diseases, lipid nanoparticles are rarely used in agricultural applications and a systematic screening approach to identifying efficacious formulations has not been reported. Here, we developed a quantitative metal-encoded platform to determine the biodistribution of different lipid nanoparticles in plant tissues. In this platform lanthanide metal complexes were encapsulated into four types of lipid nanoparticles. Our approach was able to successfully quantify payload accumulation for all the lipid formulations across the roots, stem, and leaf of the plant. Lanthanide levels were 20- to 57-fold higher in the leaf and 100- to 10,000-fold higher in the stem for the nanoparticle encapsulated lanthanide complexes compared to the unencapsulated, free lanthanide complex. This system will facilitate the discovery of nanoparticles as delivery carriers for agrochemicals and plant tissue-targeting products.

## Introduction

In modern agricultural systems, agrochemicals are critical to support different stages of crop production from seeding to growth and protection. However, it has been estimated that less than 60% of fertilizers and 0.1% of pesticides reach their target sites due to drift, runoff, and degradation^1,2,3^. The release of excess agrochemicals can also negatively interfere with inherent nutrient equilibrium in the soil and could lead to water contamination^4^. Nanotechnology has the potential to transform current crop production systems to enable precision farming to maximize output from available resources. Nano-encapsulated agrochemicals are designed to have properties to control the release of agrochemicals at target sites in response to stimuli, thus lowering application frequency and reducing ecotoxicity on the overall environment^5–10^. When guided by biorecognition ligands, targeted nanomaterials encapsulating payloads including proteins^11^, nucleotides^12^, and other bioactive molecules^13^ could enable gene editing and regulation of biological pathways related to diseases origination, nutrition deficiency, and pest control.

The broad potential of nano-agrochemicals can be attributed to their unique physicochemical properties that are compatible with and can help overcome plant barriers^14^. Stomata on the foliar epidermis is the first barrier with pores 10–30 μm long and 3–12 μm wide for gaseous exchange^13^. Nanoparticles can easily pass through these pores when they are open. At locales without the presence of stomata, the cuticle layer is the next barrier with size exclusion limits in the nanometer range. Accumulation of fluorescence-labeled nanoparticles above 50 nm was observed under the epidermis cuticle when 4–100 nm nanoparticles were applied^15^. The last and major barrier is the plant cell wall within the pore size range of 3.5–20 nm^16^. Quantum dots below such ranges were found to easily transverse through the barrier for entry into plant veins^17^. While smaller particles may diffuse through cell walls more freely, interactions between larger nanoparticles at the cell walls may create larger pores to facilitate an active transport process^18^. While size is critical, the uptake process is also dependent on the surface charge of nanoparticles since there is an unequal distribution of negative charges from the hydrophilic and hydrophobic components in the cell wall^19^. One report observed that positively charged CeO nanoparticles adhere to the root surface, while negatively charged counterparts exhibited low root accumulation^20^. Other studies suggest cell walls might promote the internalization of negatively charged gold nanoparticles rather than absorption^21^.

While metal nanoparticles have had some success, non-metal organic nanoparticles made of lipids are rarely explored in agricultural applications. Typically, lipid-based nanoparticles are composed of neutral lipids, cholesterol, an ionizable lipid, and a PEGylated lipid^22^. This multi-lipid system presents unique advantages, such as improved solubility of hydrophobic molecules, tissue-targeting localization, and better safety profiles^23^. Despite the clinical success of lipid-based nanoparticles, only a small number of reports utilize lipid nanoparticles for agrochemical delivery^24^. To date, there are no systematic approaches to determine the biodistribution of nanoparticles in plants. Inductively coupled plasma mass spectrometry (ICP-MS) is a useful approach to determine the biodistribution of nanoparticles^25^. Imaging techniques, such as X-ray, electron microscopy, and computed tomography are helpful to track the accumulation of gold nanoparticles, C70 fullerene, and carbon nanotubes at the cellular and subcellular levels. However, their application suffers from restrictive preparation protocols, analysis of only partial plant organs, and high background signals. ICP-MS, unlike previously mentioned techniques, is a mass spectrometer with the capability to simultaneously quantify multi-element samples with superior sensitivity and accuracy^26^. The uptake of liposomes containing a europium tracer into the leaf at 24 h, 48 h, and 72 h was determined by ICP-MS, suggesting a total of up to 33% of the applied dose was accumulated compared to 1% of free metal without liposome encapsulation^26^. Raliya et al.^27^ also utilized ICP-MS to explain the translocation of gold nanoparticles after an aerosolized formulation was applied to watermelon plants. The observation suggested absorption through the stomatal opening, tracked the translocation from the leaf to the root, and determined the factors related to uptake including particle shape, application approach, and plant physiology^27^.

**Figure 1:**
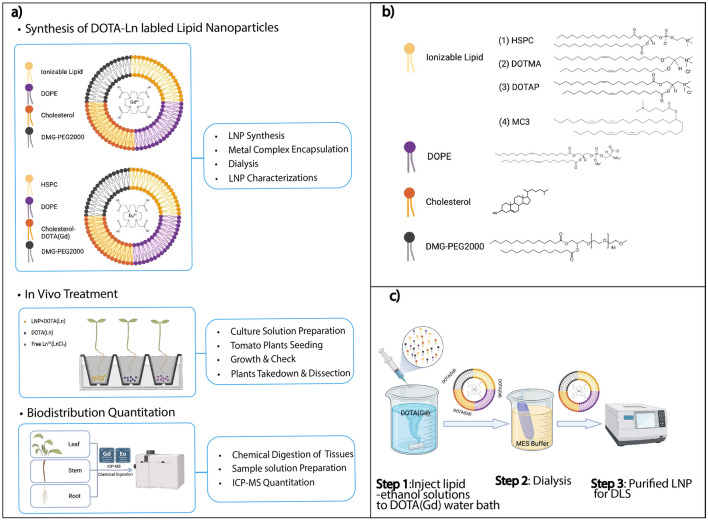
Flowchart for the development of the lanthanide-encapsulated nanoparticle platform. (**a**) Lipid nanoparticles were formulated and encapsulated with DOTA(Ln). The LNPs formulated with DOTA(Ln) were used for plant culture treatments. After incubation, the plant tissues were collected and the biodistribution was determined using ICP-MS. (**b**) Lipid nanoparticles were formulated with an ionizable lipid, structural lipid, cholesterol, and PEG lipid (chemical structures are shown). (**c**) The LNPs were prepared by mixing the lipids in ethanol and then added to the DOTA(Ln) containing aqueous phase. The resulting LNP solutions were dialyzed against MES buffer and characterized by DLS.

Here, we describe a quantitative approach to assessing nanoparticle biodistribution in plants by adapting our previously described Q-MEC protocol^28^ (Fig. 1a). As a proof of concept, we selected four lipid nanoparticles (MC3, DOTAP, DOTMA, and HSPC) that have been used either clinically and/or reported in the literature for nanoparticle-mediated delivery^29–34^(Fig. 1b). Each nanoparticle was successfully formulated with and encapsulated a DOTA(Gd) complex (Fig. 1c), a clinically utilized imaging reagent. After purification the nanoparticles were then evaluated in plants using ICP-MS to quantify the metal abundance across plant tissues. Experimental groups treated with metal-containing nanoparticles were compared to vehicle-only, free metal (no nanoparticle) control, and a DOTA(Gd) (no nanoparticle) control to determine targeting enrichment. Here, we show the utility of our quantitative metal-encoded approach as a platform to simultaneously measure the biodistribution of multiple lipid nanoparticles.

## Results

The synthesis of lipid nanoparticles was successfully accomplished using solvent-injection techniques based on optimized lipid ratios (Tables S1 and S2). A size stability test of LNPs was carried out in MES buffer and tested by DLS on Day 1 and Day 3 to match the plant culture period (Table S3). Briefly, all nanoparticles were formulated by injecting an organic phase (25 mM total lipid in ethanol) through a syringe into an aqueous phase (0.06 mg/mL DOTA-Ln in MQ water) at 70 °C under vigorous mixing conditions. The nanoparticles were then purified by dialysis to remove any unencapsulated material (Fig. S1). To characterize the nanoparticles we measured the hydrodynamic diameter (Fig. S2), polydispersity index (PDI), zeta potential, and encapsulation efficiency (Table 1). The resulting nanoparticles had an effective diameter within 200 nm and a PDI between 0.1–0.3. HSPC produced nanoparticles with the smallest diameters (94.56 nm) while DOTMA produced nanoparticles with the largest diameters (162.85 nm). The encapsulation efficiency was measured by testing the amount of DOTA(Ln) in dialysates using ICP-MS. The optimal encapsulation efficiency (Fig. S3) was obtained with HSPC nanoparticles (1.24%) while for other formulations the efficiency drops by over twofold. Even with an overall low concentration of DOTA(Ln) in the nanoparticle formulation, the signal for the biodistribution in plant tissue is still more than 1000-fold higher than the lower limit of detection by ICP-MS (1-10ppt).

**Table 1:** The size distribution, polydispersity, zeta potential, and encapsulation efficiency for the four lipid nanoparticles in MES buffer (pH 6.4): HSPC, MC3, DOTMA, and DOTAP.

Category	Effective diameter (nm)	Polydispersity	Zeta potential (mV)	Encapsulation efficiency (%)
HSPC	94.56	0.212	21.0	1.24
DOTMA	162.85	0.41	52.2	0.58
DOTAP	123.00	0.275	52.2	0.58
MC3	155.36	0.283	57.3	0.69

Zeta potential is often used to define colloidal stability by categorizing the value of ± 0–10 mV, ± 10–20 mV, ± 20–30 mV, and ± 30 mV as highly unstable, relatively stable, moderately stable, and highly stable^35^. MC3, DOTMA, and DOTAP exhibited high stability with zeta potentials above 50, while HSPC was moderately stable with a zeta potential from ± 20–30 mV. In conclusion, all nanoparticles possessed acceptable stability and reasonable size distribution, which was ideal for *in planta* experiments.

**Figure 2:**
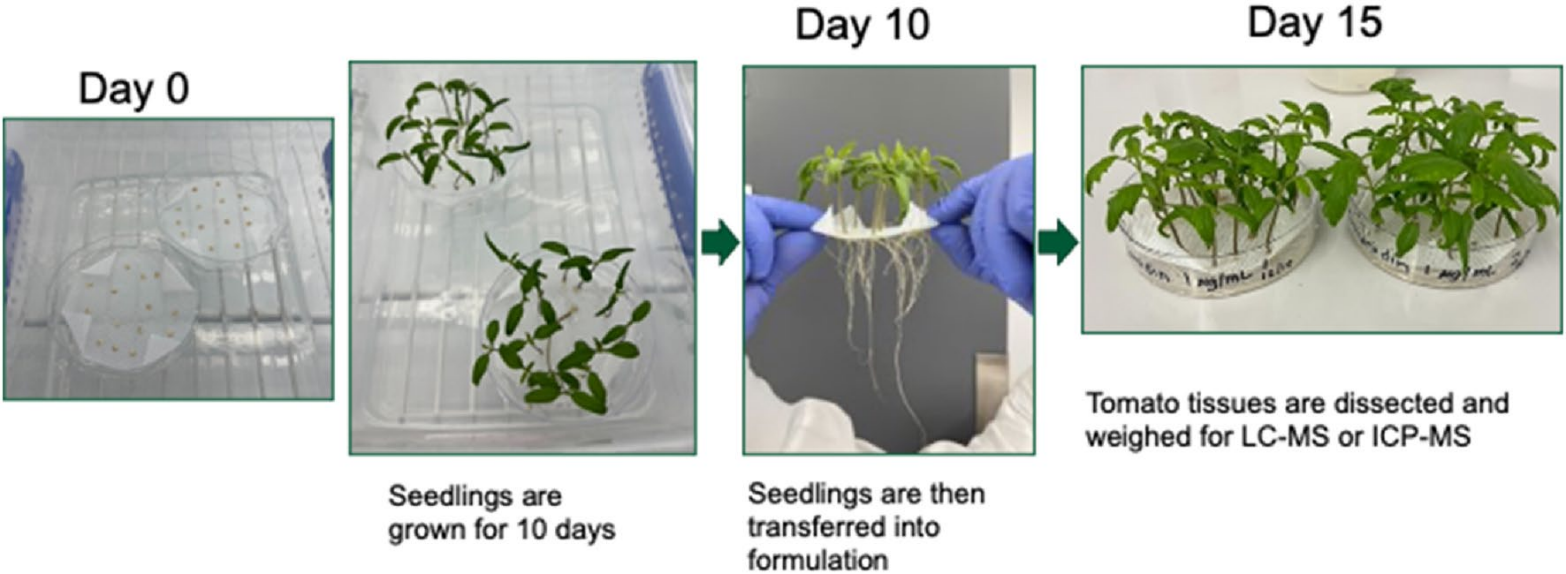
*In planta* treatment assay.

To quantitatively assess the biodistribution of the different nanoparticle formulations, we measured tissue lanthanide abundance of the administered DOTA(Gd) complex encapsulated in the four different lipid nanoparticles (HSPC, DOTMA, MC3, and DOTAP). The respective nanoparticles were dialyzed against MES buffer to remove the unencapsulated free metal complex and then administered to 10-day old tomato plant seedlings by transferring the plants into the nanoparticle-laden culture solution. After 5 days, the major plant tissues were collected (Fig. 2).

The plant tissue was then dissected into the leaf, stem, and root. After acidic digestion of the tissue, the samples were injected into an ICP-MS to quantify the lanthanide metal content. Of the three tissues, the roots of the plants exhibited the highest amount of variability (Fig. S4). The root is a major absorption tissue before the LNPs translocate to other tissues. The plants’ transport efficiency is determined by the hydraulic conductivity per cross sectional area which is proportional to the fourth power of its radius. Therefore the amount of LNPs that accumulated in the roots is determined by the vessel diameter in the roots and stem. However, the individual variation of vessel diameter might be up to 30% which might lead to large LNP accumulation variations in roots. Additionally, agrochemical accumulation in the stem and leaf is critical for whole-plant protection, so we prioritized formulations that increased biodistribution to these tissues.

**Figure 3:**
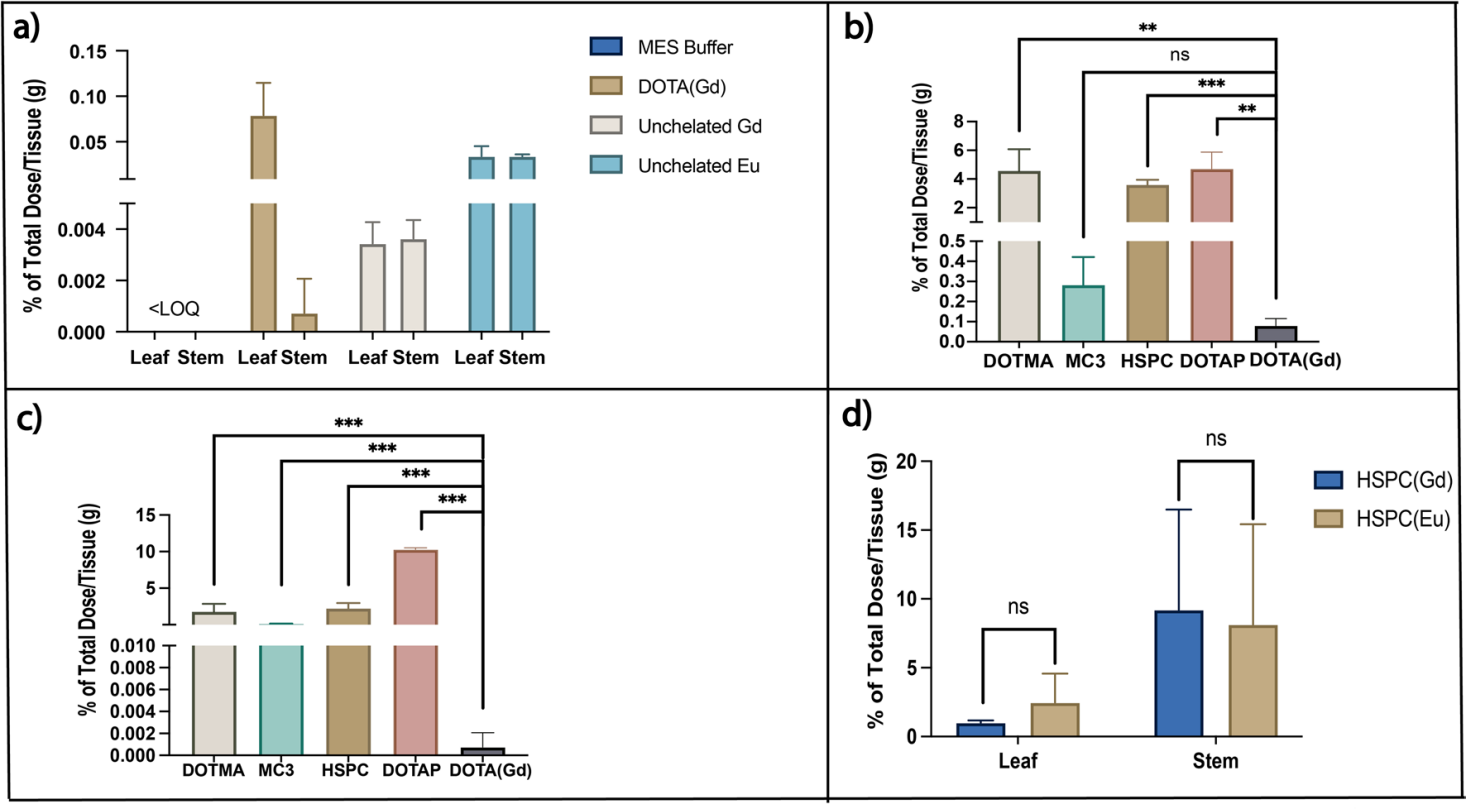
(**a**) *In planta* biodistribution after incubation with MES buffer for the metal and buffer control. (**b**) Biodistribution in leaf after incubation with MES buffer for the lipid nanoparticles formulated with DOTA(Gd). (**c**) Biodistribution in stem after incubation with MES buffer for the lipid nanoparticles with DOTA(Gd); (**d**) Biodistribution after incubation with MES buffer for the lipid nanoparticles formulated with a Gd-complex conjugated lipid and DOTA(Eu). In all cases, data are represented as mean ± SD (n = 3). All organs in the nanoparticle group were compared with the DOTA(Gd) group by Student’s *t*-test and results were labeled on the top of each column with “>” or “<” and p-value. *ns* not significant; *p ≤ 0.05; **p ≤ 0.01; ***p ≤ 0.001; ****p ≤ 0.0001.

For the non-encapsulated free Ln chloride salt or DOTA-Ln controls that were applied to the tomato plant (Fig. 3a), less than 0.1% was observed in the plant’s aerial tissues. The leaf had a higher DOTA(Gd) abundance compared to the stem, suggesting that any absorbed metal complex was transported to the leaf. Compared to the free DOTA(Gd) control, all four different nanoparticles labeled with DOTA(Gd) had significantly enhanced Gd accumulation in the leaf and stem (Fig. 3b,c). The Gd accumulation in leaf and stem was 56- and 2000-fold higher for the DOTMA formulation, respectively; 57- and 10,000-fold higher for the DOTAP formulation; 47- and 2000-fold higher for HSPC; and 20- and 100-fold higher for the MC3 formulation compared to free DOTA(Gd). The applied LNPs can help not only improve the overall accumulation in the stem and leaf, but also help diminish the accumulation difference between the leaf and stem. The biodistribution change between LNP and non-LNP encapsulated DOTA(Gd) suggests that the nanoparticles enhance payload delivery and plant uptake.

To understand if there are any differences in biodistribution between the nanoparticles and their payloads, a commercially available phospholipid conjugated to Gd-diethylenetriaminepentaacetic acid complex was included in the HSPC nanoparticle formulation as a lipid tracer, and DOTA(Eu) was encapsulated within the nanoparticles as the payload. Colocalization of both the lipid tracer and the payload in the leaf and stem would point to the importance of nanoparticle formulation in attaining the observed biodistribution, as opposed to dissociation of the payload from the particle once the nanoparticles enter the plant’s vascular system. The biodistribution of Gd and Eu in these dual metal-labeled particles were measured in tomato plants by root uptake using ICP-MS (Fig. 3d). Compared to the HSPC LNPs that were only DOTA-Gd labeled (Fig. 3b,c), double labeling HSPC LNPs indeed altered the overall accumulation in the stem, with both Gd and Eu having accumulated more in the stem than in the leaf. However, the Eu and Gd nuclei colocalized and comparable amounts of both metals were measured in both tissues, suggesting that the nanoparticles were intact and transporting their payload. Absolute accumulation of Eu and Gd in the leaf was 20-fold and 10-fold higher compared to free Eu or Gd salt respectively, which suggests that the HSPC nanoparticles impact the delivery of the payload to the leaf as well.

## Discussion

We have shown that LNPs can enhance localization *in planta* to the leaf, stem, and root. The increased localization to both the stem and leaf is particularly desirable to achieve higher distribution of agrochemicals for whole-plant protection. Colocalization of both lanthanide-labeled lipid and payload is consistent with nanoparticle formulation being necessary to impact biodistribution. Previous studies by Larue et al.^36^ using metal NPs proposed different root uptake mechanisms dependent on nanoparticle size. Larger particle diameters (> 140 nm) are unable to pass the epidermis or Casparian band in the root and are unable to transport further throughout the plant. Smaller-sized nanoparticles (< 140 nm), however, could accumulate in root parenchyma to reach the vascular cylinder and transport to the upper tissue, or pass through the epidermis cell wall directly in the root hairs and transport radially across the root cortex. Our nanoparticle formulations generally had particle size distributions above and below this cutoff and may explain why all the LNP formulations tested were able to presumably enhance transport into and across the plant. Overall, our approach to using lanthanide-labeled payload and lipid that enabled facile and quantitative determination of LNP biodistribution by ICP-MS in plants. The lanthanide-encoded approach opens an avenue for the discovery of novel nanoparticle formulations for plant delivery by directly screening unknown lipid nanoparticle collections in live plants. Based on the mass difference, we anticipate that up to 8 different lanthanide-encoded nanoparticles can be pooled at one time to enable facile characterization of tissue-targeting nanoparticles in future studies. Further elucidation of biological targets utilizing these particles with novel distribution can help targeted delivery and controlled release of critical agrochemicals. While these studies focused on direct encapsulation, it is also feasible to label particles through covalent conjugation to avoid potential metal release during translocation, enabling colocalization studies between nanoparticle excipients and payloads. To summarize, the present study highlights strategies to quantitatively determine the biodistribution of lipid nanoparticles directly in plant models by sensitive lanthanide detection. This metal-labeling approach provides a screening platform that can extend to other types of nanomaterials for the targeted delivery of agrochemicals and that can uncover new mechanisms of plant tissue targeting, eventually leading to new plant tissue-targeting products.

## Material and methods

### General information

Cholesterol was purchased from Sigma Aldrich. DOPE, DMG-PEG2000, MC3, DOTAP, DOTMA, and HSPC were purchased from Fisher Scientific. The diameter, PDI and zeta potential values of lipid nanoparticles were determined using NanoBrook Omni (Brookhaven Instruments, NY, USA). All plant studies were performed did not put any endangered species of wildlife or fauna at risk, and were performed in accordance to guidelines laid out by the “Convention on International Trade in Endangered Species of Wild Fauna and Flora” and the “IUCN Policy Statement on Research Involving Species at Risk of Extinction.”

### Statistics analysis

Quantitative data are expressed as mean ± SD from n = 3 for in vitro or n = 6 for in vivo respectively. Statistical significance between different groups was evaluated by Student’s T-test, in which *p ≤ 0.05 was considered statistically significant, and extreme significance was set as ****p ≤ 0.0001.

### Synthesis of DOTA(M)

The synthesis of DOTA(M) was described previously^28^. To the solution of Azido-mono-amide-DOTA (10 mg, 16.8 μmol) dissolved in Milli-Q water (1 mL), GdCl3 (6.6 mg, 25.2 μmol) was added. The pH of resulting solution was adjusted by 1 M NaOH to 5–6 and pH was monitored until the pH was stable > 1 h. The reaction mixture was then left stirring at 40 °C overnight. 1 M NaOH was used to adjust pH > 11 and white precipitate was formed. After stirring for 1 extra hour, the precipitate was removed by 0.2 µM PTFE filter. The unchelated metal in the filtrate was determined by xylenol orange solution. If unchelated metal was not detected, the resulting solution was concentrated by lyophilization to obtain white powder. 3 × 1 mL Ethanol was used to extract DOTA(M) after filtration from the lyophilized powder to yield white powder as final product.

### Formulation of nanoparticle

Based on the ratios in Table S1, 25 mM lipids mixtures composed of ionizable/cationic lipid, cholesterol, DOPE, and DMG-PEG2000 were dissolved in absolute ethanol at 70 °C. The MilliQ water was preheated to 70 °C. The 10% v/v lipid phase was injected via syringe into the 90% v/v aqueous phase at 70 °C, and the resulting solution was vortexed until the mixture achieved homogeneity. The nanoparticle solution was dialyzed (10,000 MWCO dialysis membrane, SpectrumLabs) against 10 mM MES buffer (pH = 5.9) and the final product was kept at 4 °C before plant experiments. To determine size distribution, homogenized solution before dialysis was diluted 10-folds against MilliQ Water for a dynamic light scattering test.

### In vivo biodistribution by metal abundance

Isolated tissues were weighed out, digested in 1 mL HNO_3_ (trace metal grade after multi-step distillation) in closed Teflon vials at 85 °C for 30 min when the mixture became homogenized and left at room temperature overnight. Following digestion, total volume for each sample was measured and adjusted into 1 mL or factored as 1 mL. A 0.64 mL aliquot was removed, diluted a 10 × with Milli-Q water to reach a 7% HNO_3_ concentration solution and then mixed with Ho as internal standard at final concentration of 1 ppb. Standards were prepared using a pure Gd, Eu, Tm, Yb and Ho standard (1 ppb Ho as internal standard) at concentrations of 0.002, 0.004, 0.006, 0.008, 0.01, 0.05, 0.1, 0.25, 0.5, 0.75, 1, 2.5, 5, 10, 25 and 50 ppb in 7% HNO_3_. During the analytical run, the blanks were run at the beginning and the samples were introduced to the instrument with standards interspersed throughout the run. Once blanks were subtracted from the signal, a calibration curve was generated by analyzing standards and this curve had R^2^ of 0.9999. Final metal concentrations were determined by comparing the signal intensity of samples from the calibration curve.

### In vivo treatment assay

#### Stage 1—preparation

Before seeding, meshes patches (6 cm × 6 cm; McMaster-Carr, Cat#1100T41) were autoclaved and 0.5 × MS media was prepared, filtered, and sterilized.

#### Stage 2—seeding

At day 0, the number of petri dishes (Fisher Scientific, Cat# FB0875711) was calculated based on the formula (# conditions x # reps + 2). Plastic containers to hold the petri dishes with plants (Sistema box, Sistema Plastics, Cat#1850) were sterilized first, soaked in 20% bleach solution for 20 min, wiped dry, sprayed with 70% ethanol including the tops, and let it dry with face down. Tomato seeds were soaked in bleach sterilization solution (75 μL bleach and 10 μL Triton X-100, diluted to 15 mL with MQ water) for 15 min with gentle agitation and rinsed with 50 mL Milli-Q water for four times. A square of sterilized mesh was placed in a petri dish and 14 sterilized tomato seeds were placed in a 3 × 4 × 4 × 3 pattern on the center of the mesh. Subsequently, 30 mL of 0.5 × MS media was carefully added under the resulting mesh. A few drops of media were placed on top of each seed to ensure contact with the media below using a micropipettor. If the liquid bubbled on top of the mesh, the seed was moved until it flowed properly. The petri dishes were placed in the Sistema boxes to which a thin layer of Milli-Q water was added to increase humidity. The boxes were covered with their tops and placed in a reach-in growth chamber (27 °C, 50% RH, 16:8 light:dark cycle). The growth was monitored on day 3, 6, 9, and media was topped off if needed.

#### Stage 3—treatment

At day 10, deep dish petri dishes were labelled and filled with 20 mL of the formulated nanoparticle solution such that the final concentration of the metal or the metal complex was at 10 nM. The empty seed coats were removed from the tomato mesh using forceps to prevent contamination. The mesh with tomato seedlings was gently removed without damaging the tomato roots from the MS media, placed on a paper towel to dry briefly, and transferred to the new petri dish with the treatment solution. The dishes were immediately placed back in the plastic box and moved back to the incubator for the 72-h incubation period.

#### Stage 4—takedown

On day 13, the mesh with tomato seedlings was gently removed without damaging the tomato roots from the treatment solution, placed on a paper towel to dry briefly, and transferred to the new petri dish with fresh Milli-Q water for rinsing. The roots were rinsed for 3 times with Milli-Q water before the true leaves, stems and roots were dissected on a paper towel and with disposable razor blades starting from the lowest active ingredient concentration. The dissected plant tissues were weighed on an analytical balance before ICP-MS analysis.

### Supplementary Information

Supplementary Information.

## Data Availability

The datasets generated during and/or analyzed during the current study are available from the corresponding author on reasonable request.
