# Supplementary material for: Quantitative biodistribution of nanoparticles in plants with lanthanide complexes
Source: Sci Rep. 2023 Dec 5;13:21440. doi: 10.1038/s41598-023-47811-4 (PMC10698154; doi:10.1038/s41598-023-47811-4)
Supplement: Supplementary file 1 — Supplementary Information. [file 41598_2023_47811_MOESM1_ESM.docx]

Quantitative Biodistribution of Nanoparticles in Plants with Lanthanide Complexes

Hou, H.^1,^**^§^**, Xu, Z.^2,^**^§^**, Takeda, Y.S.^3^, Powers, M.^3^, Yang, Y.^3^, Hershberger, K.^3^, Hanscom, Hailey.^3^, Svenson, S.^3^, Simhadri, R.K.^3^, and **Vegas, A.J.^1,2,^***

^1^Division of Materials Science and Engineering, Boston University, Boston, Massachusetts, United States

^2^Department of Chemistry, Boston University, Boston, Massachusetts, United States

^3^Invaio Sciences, Cambridge, Massachusetts, United States

**^§^ =** these authors contributed equally

* = to whom correspondence should be addressed

**Supplementary Information**

## Material and Methods

### *General Information*

Cholesterol was purchased from Sigma Aldrich. DOPE, DMG-PEG2000, MC3, DOTAP, DOTMA, and HSPC were purchased from Fisher Scientific. The diameter, PDI and zeta potential values of lipid nanoparticles were determined using NanoBrook Omni (Brookhaven Instruments, NY, USA). All plant studies were performed did not put any endangered species of wildlife or fauna at risk, and were performed in accordance to guidelines laid out by the “Convention on International Trade in Endangered Species of Wild Fauna and Flora” and the “IUCN Policy Statement on Research Involving Species at Risk of Extinction.”

**Statistics Analysis**

Quantitative data are expressed as mean ± SD from n=3 for *in vitro* or n=6 for *in vivo* respectively. Statistical significance between different groups was evaluated by Student’s T-test, in which * = p ≤ 0.05 was considered statistically significant, and extreme significance was set as **** = p ≤ 0.0001.

### *Synthesis of DOTA(M)*

The synthesis of DOTA(M) was described previously^28^. To the solution of Azido-mono-amide-DOTA (10 mg, 16.8 umol) dissolved in Milli-Q water (1mL), GdCl3 (6.6 mg, 25.2 umol) was added. The pH of resulting solution was adjusted by 1M NaOH to 5-6 and pH was monitored until the pH was stable > 1 h. The reaction mixture was then left stirring at 40 °C overnight. 1M NaOH was used to adjust pH > 11 and white precipitate was formed. After stirring for 1 extra hour, the precipitate was removed by 0.2 µM PTFE filter. The unchelated metal in the filtrate was determined by xylenol orange solution. If unchelated metal was not detected, the resulting solution was concentrated by lyophilization to obtain white powder. 3 x 1 ml Ethanol was used to extract DOTA(M) after filtration from the lyophilized powder to yield white powder as final product.

### *Formulation of Nanoparticle*

Based on the ratios in **Table S1**, 25 mM lipids mixtures composed of ionizable/cationic lipid, cholesterol, DOPE, and DMG-PEG2000 were dissolved in absolute ethanol at 70 °C. The MilliQ water was preheated to 70 °C. The 10% v/v lipid phase was injected via syringe into the 90% v/v aqueous phase at 70 °C, and the resulting solution was vortexed until the mixture achieved homogeneity. The nanoparticle solution was dialyzed against 10 mM MES buffer (pH = 5.9) and the final product was kept at 4 °C before plant experiments. To determine size distribution, homogenized solution before dialysis was diluted 10-folds against MilliQ Water for a dynamic light scattering test.

### *In vivo Biodistribution by Metal Abundance*

Isolated tissues were weighed out, digested in 1 mL HNO_3_ (trace metal grade after multi-step distillation) in closed Teflon vials at 85 ˚C for 30 min when the mixture became homogenized and left at room temperature overnight. Following digestion, total volume for each sample was measured and adjusted into 1mL or factored as 1mL. A 0.64 mL aliquot was removed, diluted a 10 X with Milli-Q water to reach a 7% HNO_3_ concentration solution and then mixed with Ho as internal standard at final concentration of 1 ppb. Standards were prepared using a pure Gd, Eu, Tm, Yb and Ho standard (1 ppb Ho as internal standard) at concentrations of 0.002, 0.004, 0.006, 0.008, 0.01, 0.05, 0.1, 0.25, 0.5, 0.75, 1, 2.5, 5, 10, 25 and 50 ppb in 7% HNO_3_. During the analytical run, the blanks were run at the beginning and the samples were introduced to the instrument with standards interspersed throughout the run. Once blanks were subtracted from the signal, a calibration curve was generated by analyzing standards and this curve had R^2^ of 0.9999. Final metal concentrations were determined by comparing the signal intensity of samples from the calibration curve.

***In vivo* Treatment Assay**

**Stage 1 - Preparation:** Before seeding, meshes patches (6 cm x 6 cm; McMaster-Carr, Cat#1100T41) were autoclaved and 0.5x MS media was prepared, filtered, and sterilized.

**Stage 2- Seeding:** At day 0, the number of petri dishes (Fisher Scientific, Cat# FB0875711) was calculated based on the formula (# conditions x # reps + 2). Plastic containers to hold the petri dishes with plants (Sistema box, Sistema Plastics, Cat#1850) were sterilized first, soaked in 20% bleach solution for 20 min, wiped dry, sprayed with 70% ethanol including the tops, and let it dry with face down. Tomato seeds were soaked in bleach sterilization solution (75 μL bleach and 10 μL Triton X-100, diluted to 15 mL with MQ water) for 15 minutes with gentle agitation and rinsed with 50 mL Milli-Q water for four times. A square of sterilized mesh was placed in a petri dish and 14 sterilized tomato seeds were placed in a 3x4x4x3 pattern on the center of the mesh. Subsequently, 30 mL of 0.5x MS media was carefully added under the resulting mesh. A few drops of media were placed on top of each seed to ensure contact with the media below using a micropipettor. If the liquid bubbled on top of the mesh, the seed was moved until it flowed properly. The petri dishes were placed in the Sistema boxes to which a thin layer of Milli-Q water was added to increase humidity. The boxes were covered with their tops and placed in a reach-in growth chamber (27 ºC, 50% RH, 16:8 light:dark cycle). The growth was monitored on day 3, 6, 9, and media was topped off if needed.

**Stage 3 – Treatment:** At day 10, deep dish petri dishes were labelled and filled with 20 mL of the formulated nanoparticle solution such that the final concentration of the metal or the metal complex was at 10 nM. The empty seed coats were removed from the tomato mesh using forceps to prevent contamination. The mesh with tomato seedlings was gently removed without damaging the tomato roots from the MS media, placed on a paper towel to dry briefly, and transferred to the new petri dish with the treatment solution. The dishes were immediately placed back in the plastic box and moved back to the incubator for the 72-hour incubation period.

**Stage 4 – Takedown:** On day 13, the mesh with tomato seedlings was gently removed without damaging the tomato roots from the treatment solution, placed on a paper towel to dry briefly, and transferred to the new petri dish with fresh Milli-Q water for rinsing. The roots were rinsed for 3 times with Milli-Q water before the true leaves, stems and roots were dissected on a paper towel and with disposable razor blades starting from the lowest active ingredient concentration. The dissected plant tissues were weighed on an analytical balance before ICP-MS analysis.

**Supplemental Data**

| Molar ratio | Ionizable/cationic lipid | DOPE | Cholesterol | DMG-PEG2000 |
| --- | --- | --- | --- | --- |
| MC3 | 50 | 10 | 38.5 | 1.5 |
| DOTAP | 44 | 44 | 12 | - |
| DOTMA | 44 | 44 | 12 | - |
| HSPC | 60 | - | 38 | 2 |

**Table S1.** The optimized lipid ratios used for Nanoparticle formulation.

| HSPC | Glucose (Y/N) |  |  |
| --- | --- | --- | --- |
|  | Y | 186.8 | 0.35 |
|  | N | 153.4 | 0.23 |
|  | Lipid Concentration (mM) |  |  |
|  | 50 mM | 160.19 | 0.215 |
|  | 25 mM | 123.15 | 0.232 |
|  | 10 mM | 157.17 | 0.174 |
|  | Temperature (ºC) |  |  |
|  | 65 | 123.15 | 0.232 |
|  | 70 | 111.23 | 0.260 |
|  | 70 (injection through syringe) | 95.68 | 0.233 |
| DOTMA | Glucose (Y/N) |  |  |
|  | Y | 106.1 | 0.41 |
|  | N | 100.2 | 0.33 |
|  | State of lipid |  |  |
|  | Powder | 106.1 | 0.41 |
|  | Chloroform | 347.66 | 0.31 |
|  | Chloroform evaporated | 177.53 | 0.154 |
| DOTAP | Glucose (Y/N) |  |  |
|  | Y | 124.5 | 0.5 |
|  | N | 83.3 | 0.26 |
|  | State of Lipid |  |  |
|  | Chloroform | 123.00 | 0.275 |
|  | Powder | 178.86 | 0.152 |

**Table S2.** Parameter optimization for the formulation of HSPC, DOTMA and DOTAP nanoparticles.


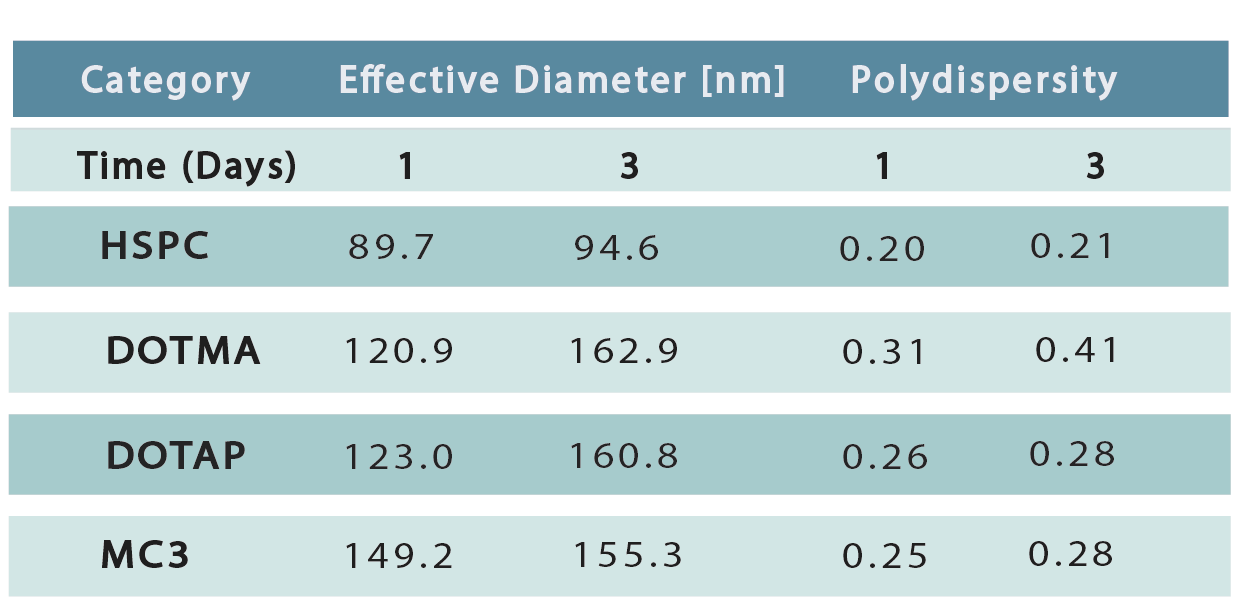


**Table S3.** Particle size and polydispersity of lipid nanoparticles in MES buffer (pH 6.4) at Day 1 and Day 3.


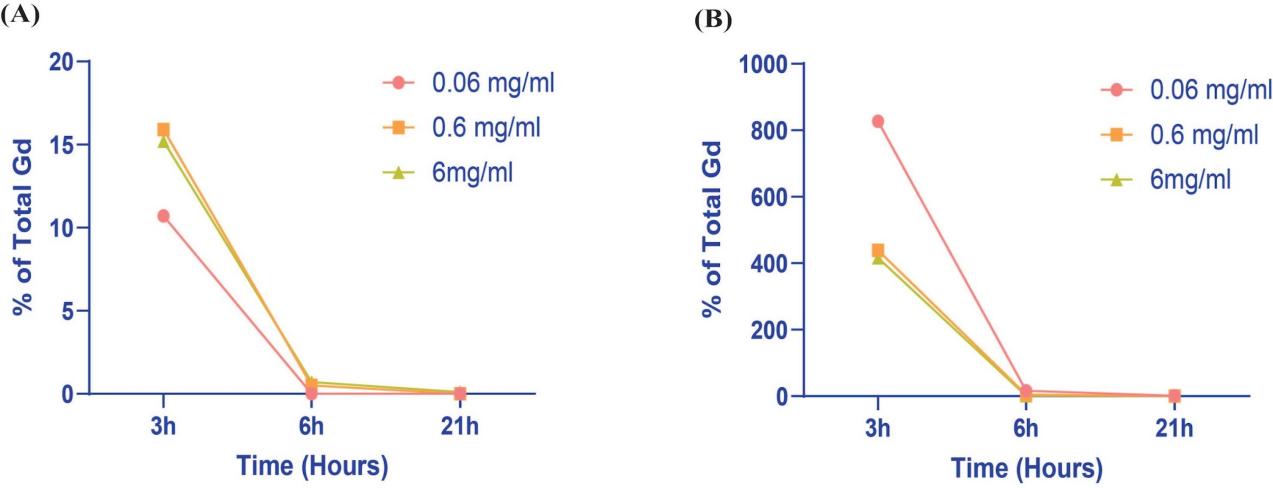
**Figure S1.** The percentage of Gd in MES dialysate measured by ICPMS at 3^rd^, 6^th^ and 21^st^ hour for the HSPC nanoparticles from (A) organic encapsulation; (B) water encapsulation.


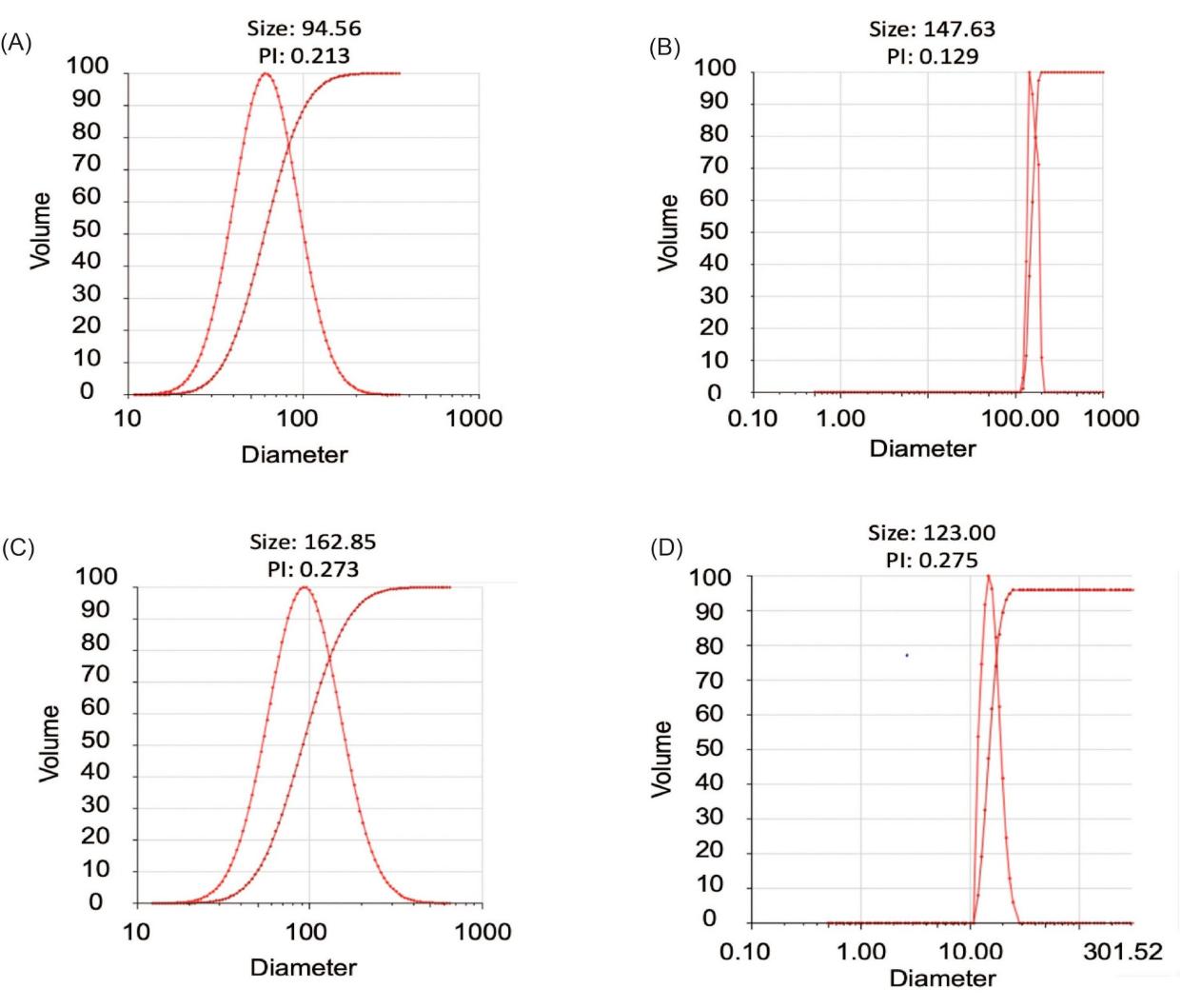


**Figure S2.** The size distribution is measured by dynamic light scattering for the lipid nanoparticles, (A) HSPC; (B) MC3; (C) DOTMA; (D)DOTAP by size and polydispersity index (PI).


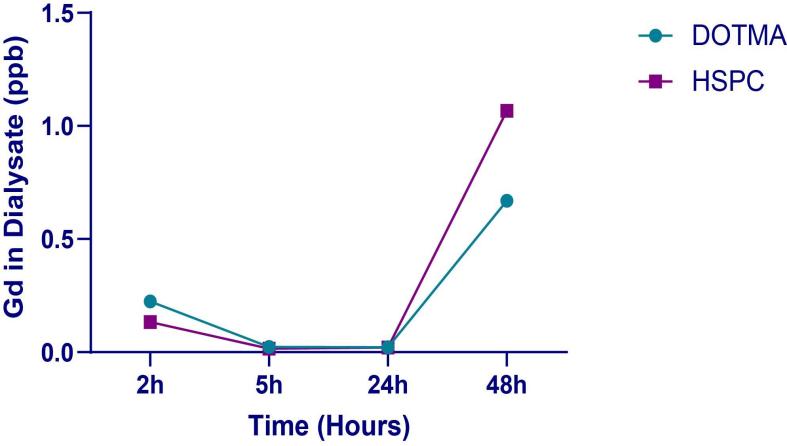
**Figure S3.** The encapsulation stability of DOTMA and HSPC nanoparticles measured by ICPMS.


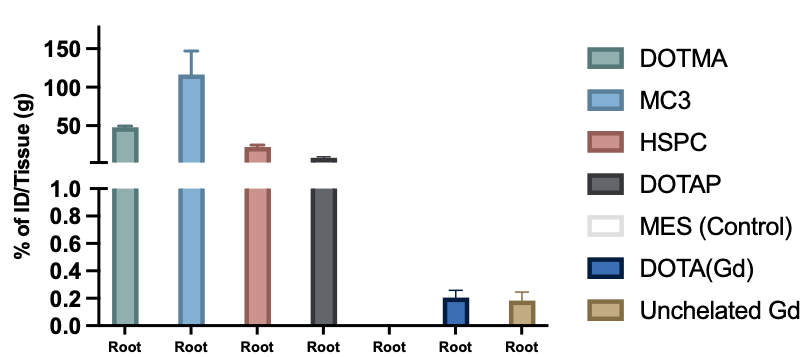


**Figure S4.** In planta biodistribution for tomato plant roots
